# Evaluation of Silkworm Cocoon-Derived Biochar as an Adsorbent for the Removal of Organic and Inorganic Contaminants from Rainwater

**DOI:** 10.3390/ma18215053

**Published:** 2025-11-06

**Authors:** Anna Marszałek, Ewa Puszczało, Mariusz Dudziak, Anna Pajdak, Jakub Frankowski

**Affiliations:** 1 Faculty of Energy and Environmental Engineering, Silesian University of Technology, Konarskiego 18, 44-100 Gliwice, Poland; ewa.puszczalo@polsl.pl (E.P.); mariusz.dudziak@polsl.pl (M.D.); 2 Strata Mechanics Research Institute, Polish Academy of Sciences, Reymonta 27, 30-059 Kraków, Poland; pajdak@imgpan.pl; 3 School of Medical and Health Sciences, VIZJA University, Okopowa 59, 01-043 Warszawa, Poland; j.frankowski@vizja.pl

**Keywords:** heavy metals, benzotriazole, adsorption kinetics, Langmuir and Freundlich isotherms, BET surface area

## Abstract

This study presents evaluation of biochar derived from silkworm cocoons for the adsorption of organic and inorganic contaminants from rainwater. The material was characterised using BET surface area analysis, scanning electron microscopy (SEM), and the point of zero charge (pH_PZC_). The prepared biochar exhibited a well-developed surface area and demonstrated adsorption capacity toward both heavy metals and benzotriazole. The model rainwater was prepared by spiking real rainwater samples with Cu(II), Ni(II), Zn(II) ions, and benzotriazole (BT). Adsorption experiments were carried out under laboratory conditions to evaluate the effects of contact time, pH, and sorbent dosage. The experimental data were fitted to pseudo-first-order and pseudo-second-order kinetic models, as well as Langmuir/and Freundlich isotherms. The results showed that the adsorption of Cu(II) followed the Langmuir/Freundlich model, while the adsorption of Ni(II) benzotriazole was more consistent with the Freundlich model. Adsorption kinetics were best described by the pseudo-second-order model. The highest removal efficiencies were observed for Cu(II) (96%) and Ni(II) (88.8%), while Zn(II) removal was limited. Benzotriazole was also effectively adsorbed (97%), rapid adsorption occurred mainly within the first minute. Overall, the study highlights the selective adsorption behaviour of silkworm cocoon biochar and provides a comparative insight into the removal of organic and inorganic pollutants using a waste-derived adsorbent with surface properties comparable to those of activated carbon.

## 1. Introduction

Rainwater, although often perceived as naturally clean, can contain a variety of organic and inorganic pollutants from human activities and environmental processes [1]. Among the most common inorganic substances are heavy metals such as copper, zinc, nickel and lead, which end up in rainwater as a result of industrial emissions, road transport, and the use of construction materials [2]. Zinc, often derived from road dust, tyres, and lubricants, is highly soluble in water, while copper from similar sources has moderate solubility. Nickel, released through industrial activities, energy production, and vehicle emissions, can also contaminate rainwater [3,4,5]. Benzotriazole, an organic pollutant with limited water solubility, originates primarily from corrosion inhibitors in coolants and brake fluids and can reach rainwater through leaks, industrial dust or indirect wastewater [6]. Their presence in rainwater poses a serious threat to aquatic ecosystems and human health. Untreated rainwater can pollute rivers, lakes, and waters, leading to the degradation of aquatic ecosystems and access to clean drinking water [7].

The adsorption process with the use of biochar is one of the most effective methods for the removal of organic and inorganic pollutants from water [8,9]. A novel approach is the use of biological waste raw materials, such as the mulberry silkworm cocoon, as a raw material for biochar production. To complete the life cycle, the caterpillar forms a cocoon after five stages of development. During the breeding process, while the pupa has formed, the cocoon is cut. After the butterflies hatch, the cut cocoon cannot be used for textile purposes. Hence, it is an organic waste that can be used as a water purification filter [10].

The silkworm cocoon, due to its unique chemical composition and fibre structure, can be an excellent base for the production of high-performance adsorption materials [11]. The cocoon consists of two types of proteins: fibroin, the main structural element that accounts for approximately 70% of the weight of the cocoon, and sericin, soluble in hot water, which bonds the fibroin filaments to each other and accounts for approximately 30% [12]. After the removal of sericin and subsequent functionalisation, the silkworm cocoon becomes a biodegradable adsorbent with high efficiency in the removal of heavy metal ions and dyes from water. Researchers have shown that functionalised silk composites achieve adsorption efficiencies exceeding 90% for copper ions, 93.75% for lead ions, and adsorption capacities of 88.5 mg/g for acid yellow 11 and 74.63 mg/g for naphthol orange, confirming their excellent performance in pollutant removal [13]. Silk is one of these natural and biodegradable materials with good biocompatibility, mechanical strength, and nontoxicity [12]. For example, Pilley et al. demonstrated that silk fibroin is a nontoxic, economical, and eco-friendly adsorbent with exceptional efficiency (12.82 mg/g) and high iron removal efficiency (98%), when used for up to 5 cycles [14]. Natural silk fibroin powder can serve, for example, as an excellent adsorbent for the removal of various metal ions, including Cu(II), Cd(II), Ni(II) from aqueous solutions [15]. Sericin powder obtained from silkworm cocoons also shows potential for use as a biosorbent in the treatment of wastewater containing Bordeaux S dye [16]. Several studies have been found that focus on the use of silkworm cocoons for the production of biochar [17,18,19]. For example, Sun et al. [17] synthesised nitrogen-doped porous carbon from the silkworm cocoon and achieved a high specific surface area of 3134 m^2^/g, with an adsorption capacity for chrome of 366.3 mg/g. Xu et al. [18] prepared N-doped porous carbon for selective separation of zinc ions, reporting a BET surface area of the BET of 1994 m^2^/g. ElShafei et al. [19] used activated carbon derived from silkworm faces with a surface area between 1000 and 2000 m^2^/g for the removal of cadmium and methylene blue. Although biochar derived from protein precursors such as sewage sludge [20], microalgae [21], and biomass [22] has also been appreciated and widely studied, the utilisation of silkworm cocoon waste remains under research. This study highlights the value of this unique biological waste, offering a sustainable and effective alternative for the production of high-performance adsorbents.

The main objective of the present study is to investigate the adsorption potential of biochar derived from silkworm cocoons. This study presents an evaluation of the adsorption of heavy metals (copper, nickel, and zinc) and benzotriazole on the biochar. Additionally, information was provided about the porous structure and surface charge. An evaluation was conducted to assess the influence of the dose of adsorbent, contact time, and solution pH on the contaminant removal efficiency, in addition to analysis of adsorption isotherms and reaction kinetics to understand the adsorption mechanisms. The findings provide a basis for evaluating the applicability of this biochar in rainwater treatment and potentially in the purification of other types of contaminated water, such as industrial wastewater, greywater, or landfill leachate. This study demonstrates that silkworm cocoon biochar offers a selective and efficient adsorption of both inorganic and organic contaminants under conditions that mimic rainwater, distinguishing it from previous studies focused solely on single contaminant systems or synthetic solutions.

## 2. Materials and Methods

### 2.1. Preparation of Biochar

**Figure 1:**
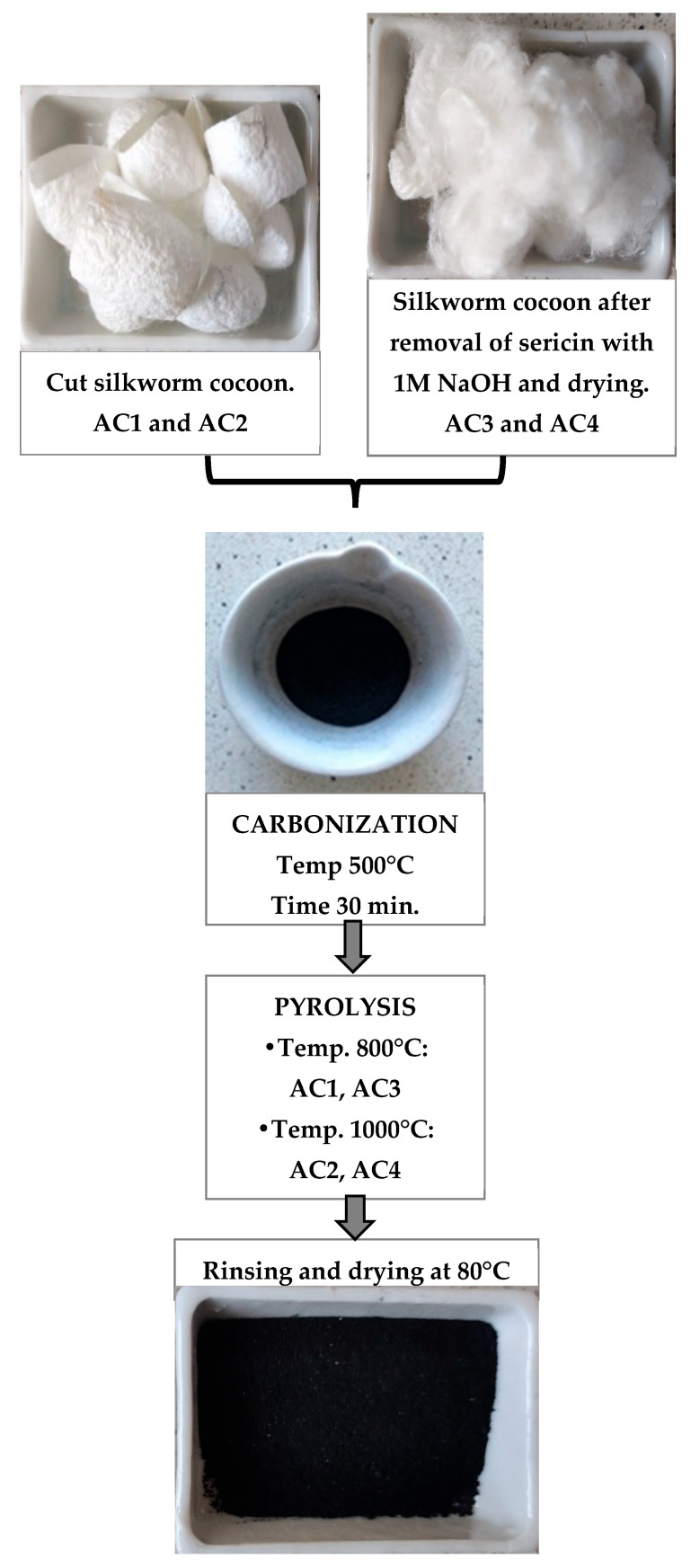
Biochar preparation flow chart.

Four biochar samples were prepared from the waste silkworm cocoon (Figure 1). Biochar was prepared in two ways:

1. The cut cocoon was carbonised directly in a muffle furnace at 500 °C for 30 min. The precarbonised product was then mixed with potassium hydroxide (KOH 1 M) in a mass ratio and then activated at 800 °C (AC1) and 1000 °C (AC2) in a tube furnace under a nitrogen atmosphere. Pyrolysis duration 2 h, with a ramp rate of 10 °C/1 min. After being cooled to room temperature, the product was washed with a 4% aqueous solution of hydrochloric acid (HCl) and then rinsed several times with deionised water to neutral pH. The mixture was then dried in a drying oven at 80 °C.
2. The cut cocoon was immersed in a 1 M NaOH solution, with 20 g of cocoon placed in 1 L of the solution. They stirred with a glass baguette at 25 °C for about an hour, checking the cocoon condition every 10 min, and hydroxycin was hydrolysed, exposing the fibroin fibres. With moderate time and temperature, the fibroin will remain intact. The solution became cloudy and the fibres separated. After the NaOH action was complete, the fibres were immediately rinsed with a large amount of distilled water to remove the residual base. The separated fibres were dried at 60 °C for 12 h. Then the procedure was followed as in the case of point 1. carbonised and pyrolyzed under the same conditions. The samples were named AC3 (800 °C) and AC4 (1000 °C).

### 2.2. Chemical Analysis

The chemical composition of the collected rainwater was examined by measuring various parameters, including chemical oxygen demand (COD), total organic carbon (TOC), heavy metals (Pb, Zn, Ni, Cu), hardness, pH, and electrical conductivity. COD and heavy metal concentrations were determined using spectrophotometric methods with Merck Spectroquant^®^ test kits (Pharo 100, Darmstadt, Germany). TOC levels were quantified using a TOC-L analyser (Shimadzu, Kyoto, Japan), while pH and conductivity were monitored with a multifunctional CPC-505 device (Elmetron, Zabrze, Poland). Analytical reagents such as nitric acid (HNO_3_) and sodium hydroxide (NaOH) were supplied by Sigma Aldrich (Poznań, Poland).

Benzotriazole (BT) in the samples was analysed using high-performance liquid chromatography (HPLC, Agilent Technologies, Santa Clara, CA, USA) equipped with a Gold Hypersil C18 column (250 × 4.6 mm, particle size 5 μm) and a diode-array detector (DAD). Prior to HPLC, BT was extracted from water samples via solid-phase extraction (SPE) using C18 cartridges (Supelclean™ ENVI, Sigma-Aldrich, Poznań, Poland). The cartridges were first conditioned with methanol, acetonitrile, and water. Five millilitres of sample was passed through the cartridge, which was then dried and eluted with 1.5 mL of a methanol–acetonitrile mixture of methanol—acetonitrile (60:40, *v*/*v*). The resulting extracts were analysed at a detection wavelength of 275 nm.

### 2.3. Characteristics of Rainwater

In the adsorption process, rainwater was treated from the roof of a single-family house located in the Silesian Voivodeship in the city of Tychy. Near the place where the water was taken there is the Civic Brewery, the National Road DK-44, and two gas stations. The rainwater was characterised by a high COD value of 108 mg/L and an organic carbon content of 16.21 mg/L. The water colour was 37 mgPt/L. pH 7.2 and conductivity was 367 μS/cm. Among heavy metals, a high concentration of zinc was observed at 1.74 mg/L. The results are presented in Table 1, which shows the physicochemical characteristics of the rainwater samples collected immediately after rainfall.

The model water was prepared using collected rainwater. Two types of rainwater were prepared: one spiked with heavy metals (Cu(II), Zn(II), Ni(II)) and one with benzotriazole (BT). Metal concentrations were adjusted to 4 mg/L each and BT to 1 mg/L. These concentrations were deliberately chosen to allow detailed evaluation of biochar adsorption properties, including fitting of data to Langmuir and Freundlich isotherms and kinetic models. At natural environmental concentrations, nearly complete removal would occur even at very low adsorbent doses, precluding reliable model fitting. Thus, the selected concentrations provide meaningful insights into the sorption mechanisms and performance under controlled conditions, while future studies will assess adsorption at more realistic environmental levels and in dynamic systems.

### 2.4. Characteristics of the Resulting Biochar

The pore structure of the samples was characterised by the volumetric low-pressure nitrogen adsorption (LPNA) method on the ASAP 2020 analyser (Micromeritics, Norcross, GA, USA). Nitrogen adsorption isotherms were determined under controlled pressure conditions in the range of 0–0.1 MPa and a temperature of 77 K. Based on nitrogen isotherms, the specific surface area was determined assuming a monolayer Langmuir model [23] and a multilayer BET model [24] filling of adsorbate molecules, the mesopore volume BJH model [25], the micropore volume DFT model [26] and the pore size distribution (DFT model).

The surface topography of the samples was determined using the SEM method using a JSM 6360LA scanning electron microscope (JEOL, Tokyo, Japan). Images were recorded at 1000× magnification with an acceleration voltage of 20 kV and an HPV peak width of 414 µm and a WD distance of 10.6 mm, allowing high image resolution and good contrast to be obtained.

The point of zero charge (pH_PZC_) for adsorbent AC3 was determined using the suspension method. In brief, 100 mg of each adsorbent was added to 100 mL of distilled water with initial pH values ranging from 2 to 10, and the suspensions were stirred at 25 °C for 24 h. After equilibration, the final pH (pHf) of each solution was recorded. The pH_PZC_ corresponds to the pH at which the plot of ΔpH = (pHf − pHi) versus pHi intersects the zero line. The initial pH values were adjusted with 0.1 M HNO_3_ or NaOH solutions [27].

### 2.5. Adsorption Process

Adsorption experiments were conducted in batch mode at room temperature to evaluate the effects of contact time, adsorbent dose, and solution pH on the adsorption capacity. All tests were performed in 100 mL glass Erlenmeyer flasks placed on an incubator shaker operating at 300 rpm.

#### 2.5.1. Effect of Adsorbent Dose

For the dose-effect studies, 50 mL of model solution was used with adsorbent doses ranging from 1 to 6 g/L. The initial pH of the collected rainwater was approximately 7.2 and was maintained without adjustment to simulate natural conditions.

#### 2.5.2. Kinetic Studies

Kinetic experiments were carried out by adding 0.15 g of adsorbent to 50 mL of the model solution containing all three metals (Cu(II), Zn(II), and Ni(II)) simultaneously, also to a separate solution containing only benzotriazole (BT). The mixtures were agitated at 300 rpm for predetermined time intervals: 5, 10, 20, 30, 60, 120, and 180 min for the metals, and up to 180 s for the BT. The initial pH during the kinetic experiments was maintained at 7.2. At each time point, the adsorbent was separated from the liquid phase for analysis.

#### 2.5.3. Effect of pH

In pH-dependent adsorption studies, the pH of the solution was adjusted to 2, 4, 6, 8 and 10 using 0.1 M HNO_3_ or NaOH solutions. For metals, the adsorbent dose was 0.1 g per 50 mL of solution, with a sorption time of 60 min. For BT, the adsorbent dose was 0.05 g per 50 mL of solution, with a sorption time of 180 s.

Real rainwater, collected immediately after rain and without any prior treatment, was used as the base solution for all experiments to reflect realistic environmental conditions and ensure the practical applicability of the studied adsorption process.

### 2.6. Models of Adsorption Process Description Used

Various models of adsorption and adsorption kinetics can be used for the analysis of adsorption on biochar. To determine adsorption, the experimental data was analysed using pseudo-first order and pseudo-second-order kinetic models, which have been described in Kamińska and Bohdziewicz, 2016 [27]. The experimental data were fitted by two common adsorption isotherm models: The Langmuir model and the Freundlich model [28]. Then, to evaluate the rate control mechanism, the experimental data were fitted using a Weber-Morris intraparticle diffusion model, which describes the article by Marszałek et al., 2022 [29].

### 2.7. Statistical Analysis of the Results

The results presented in the figures and tables reflect the arithmetic means obtained from three independent experimental repetitions. The removal efficiency was calculated using Equation (1):

Removal degree (%) = ((C_0_ − C)/C_0_) × 100% (1)

where C_0_ is the initial concentration of the compound (mg/L), and C is the concentration after adsorption (mg/L).

Experimental variability is expressed as the standard error of the mean (SE), calculated as:

SE = σ/√n(2)

where σ is the standard deviation of the measurements, and n is the number of repetitions. The error bars shown in the figures represent SE.

## 3. Results and Discussion

### 3.1. Characteristics of Biochar

**Figure 2:**
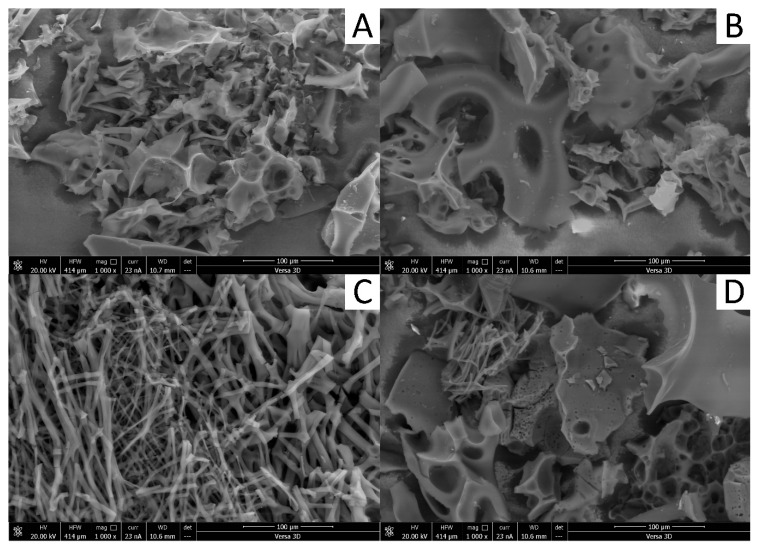
Scanning electron microscopy (SEM) of biochar from a carbonised silkworm cocoon in AC1 (**A**) and AC2 (**B**), AC3 (**C**), and AC4 (**D**).

To analyse the surface topography of the samples, scanning electron microscope (SEM) images were obtained (Figure 2).

The SEM images (Figure 2) illustrated the structure of biochar samples, revealing pores and slits ranging in size from a few to several dozen micrometres. The particles appeared as irregular sheets with cylindrical pores or elongated trabeculae and fibres. In the AC1 and AC2 samples (Figure 2A,B), which were treated with KOH (1 M) during synthesis, a mixture of sheet-like structures was observed, exhibiting varying sizes and irregular edges. These edges served as potential reactive sites for chemisorption processes. The AC3 sample (Figure 2C) differed significantly from the others in terms of microporous structure. It consisted of fragmentarily connected trabeculae and fibres, which form a network of slit-like pores with a potentially large contact surface for adsorbate interactions. In the AC4 sample (Figure 2D), very small cylindrical pores were visible within the sheet structure, along with well-developed fibres, similar to those in AC3. The presence of fibres in both AC3 and AC4 was the result of a similar synthesis pathway for this biochar. The differences in pore structure and surface topography are closely related to KOH treatment and activation temperature. AC3 (800 °C) shows slit-like micropores formed by optimal activation of KOH, while AC4 (1000 °C) shows predominantly cylindrical pores due to partial collapse at higher temperatures. This shows that both the chemical activation and temperature strongly influence the pore geometry and overall adsorption performance.

**Figure 3:**
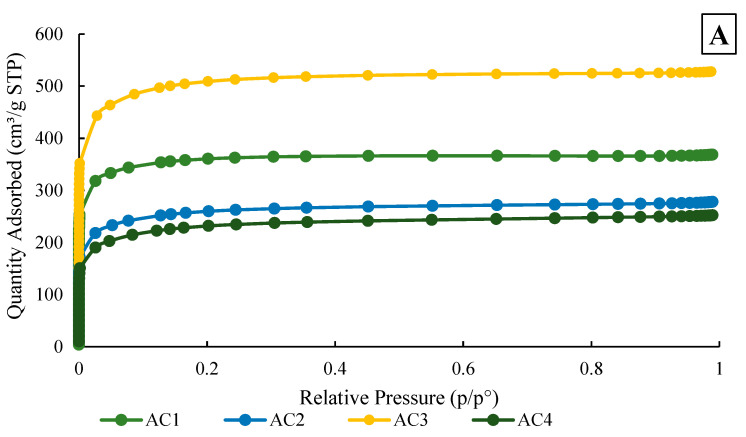

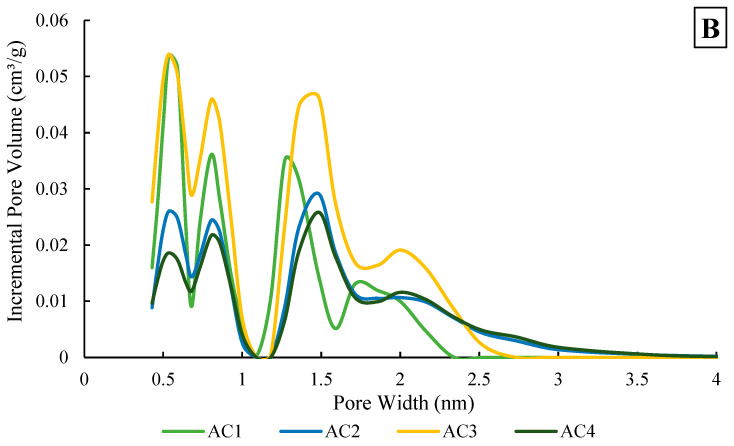
Nitrogen adsorption–desorption isotherms of AC biochars (**A**) and pore size distribution calculated using the density functional theory (DFT) model (**B**).

As a result of LPNA structural studies in biochar, nitrogen adsorption isotherms were obtained in a relative pressure range of 0 < p/p^0^ < 0.996 (Figure 3). According to the IUPAC classification [30], all isotherms were identified as type I. The most significant increase in adsorption capacity occurred at low relative pressures (0 < p/p^0^ < 0.02), after which the isotherms gradually levelled off. The N_2_ sorption capacity at a pressure of 0.1 MPa was 368 cm^3^/g and 278 cm^3^/g for AC1 and AC2, respectively. The highest sorption capacity was observed for sample AC3, reaching 528 cm^3^/g, while for AC4, it was 252 cm^3^/g.

Based on the sorption capacity data as a function of pressure, the structural parameters of the biochar samples were determined using theoretical models (Table 2). The highest specific surface area values were obtained using the Langmuir model, which accurately described the shape of the isotherms (Figure 3). The results confirm the SEM image observations, highlighting differences in the structure and adsorption properties of the synthesised carbons depending on the activation temperature. The monolayer specific surface area of the AC1 and AC2 was 1610 m^2^/g and 1198 m^2^/g. At the same time, the specific surface area, assuming multilayer surface adsorption, was 1178 m^2^/g and 856 m^2^/g, respectively. An activation temperature of 800 °C proved to be more optimal. The AC1 sample exhibited a higher micropore volume (DFT model) of 0.48 cm^3^/g, while the AC2 sample had a larger mesopore volume (BJH model) of 0.09 cm^3^/g. For the AC3 and AC4 samples, the degree of development of the pore structure was strictly dependent on the activation temperature. The highest Langmuir-specific surface area was observed in the sample activated at 800 °C (AC3), reaching 2303 m^2^/g. These results are consistent with other studies that indicate that KOH activation at temperatures around 800 °C is optimal for obtaining high degrees of porosity and surface area in carbon materials [31]. Activation at 1000 °C resulted in a lower specific surface area of 1083 m^2^/g. This trend was also reflected in the pore volume: after activation at 800 °C, the AC3 sample had a micropore volume of up to 0.68 cm^3^/g, while activation at 1000 °C (AC4) led to a significantly lower micropore volume of 0.123 cm^3^/g. A similar, though less pronounced, effect was observed in the mesopore volume. For example, Vashchynskyi et al. showed that as the temperature increases to 900 °C, the narrow micropores degenerate and the carbon matrix transforms, resulting in a decrease in both the total area and the total pore volume [32]. Therefore, careful control of the activation temperature is essential to maximise the adsorption efficiency by maintaining a large surface area and a well-developed microporous structure.

The pore size distribution was determined using the DFT model (Figure 3). All tested samples exhibited a bimodal pore size distribution, with distinct peaks in both the micropore and narrow mesopore ranges. This bimodality is a typical feature of KOH-activated biochar and arises from the two-step development mechanism of the porous structure. Initially, micropores are created during the reaction between KOH and the carbon matrix, forming narrow channels. As the activation temperature increases, partial pore widening and wall collapse occur, leading to the development of mesopores, especially around 800 °C [33]. Furthermore, the intrinsic structure of the silkworm cocoon precursor, composed of proteins and semi-crystalline components, may also promote the formation of pores in multiple size ranges. This hierarchical porosity enhances the adsorption performance of the material, particularly under flow conditions [34,35].

In all carbons synthesised, peaks with increased volume peaks were observed in the micropore range, corresponding to pore diameters of approximately 0.5 nm, 0.8 nm and 1.3–1.5 nm. These characteristics are particularly advantageous in environmental applications, such as water purification, where both high surface area and hierarchical porosity are desired [36]. According to the IUPAC classification, these materials were identified as microporous. The largest pore volume was found in the samples activated at 800 °C (AC1, AC3). In sample AC1, the most developed micropores had a diameter of 0.5 nm, whereas in sample AC3, both 0.8 nm micropores and pores on the border of micro- and mesopores had a large volume. The more widely developed pore structure in the samples activated at 800 °C may be advantageous for water purification processes, particularly in the removal of heavy metal ions. On the contrary, the pore volume in the samples activated at 1000 °C (AC2, AC4) was lower and their pore size distribution was very similar. This suggests that the higher activation temperature (1000 °C) may have been excessive, leading to partial collapse or closure of previously formed pores.

The specific surface areas of silk cocoon–derived carbons reported in the literature vary widely depending on the activation and doping conditions. For example, Sun et al. (2017) [17] obtained a very high surface area of about 3134 m^2^/g for a nitrogen-doped hierarchical porous carbon activated with KOH, where the additional nitrogen doping promotes extensive micropore and mesopore formation. Similarly, Kong et al. (2022) [35] reported 1827.9 m^2^/g for a nitrogen-doped porous carbon derived from a silk cocoon, confirming the strong influence of activation and doping on pore development. On the contrary, studies using less extensive activation or precursors based on fibroin and sericin generally show lower surface areas, typically below 1000 m^2^/g [14,16,18]. These comparisons indicate that KOH activation alone, without additional nitrogen doping, can still effectively produce a hierarchical porous structure, while additional nitrogen incorporation can further enhance pore development and surface area.

**Figure 4:**
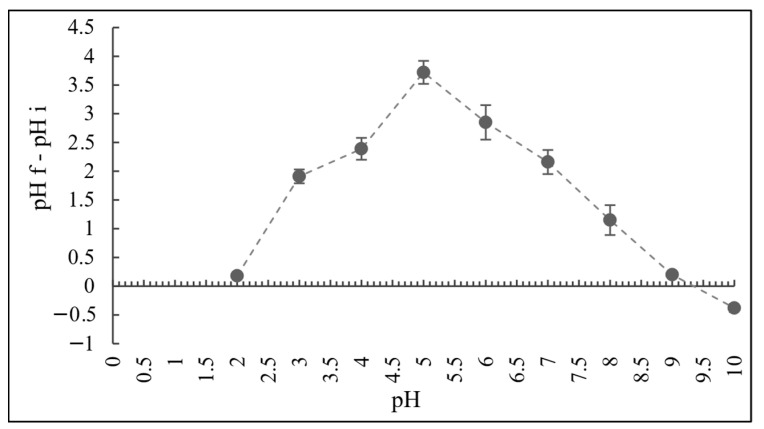
Determination of the surface charge zero point for the AC3 sample, mean ± SD (n = 3).

These structural advantages are consistent with the measured high pH_PZC_ of AC3 (9.3), indicating a strong alkaline surface (Figure 4). The value obtained falls within the range commonly reported for KOH-activated biochar (8–11) [37] and is higher than that of many unmodified biochar (5–7) [38], suggesting that KOH activation increases the surface basicity of the material, in agreement with previous literature findings. The high pH_PZC_ suggests that the biochar surface is predominantly negatively charged at neutral and acidic pH values, which favours the adsorption of positively charged metal ions.

Furthermore, KOH acts as a chemical activating agent that enlarges the pore network and increases the specific surface area of biochar. During activation, gaseous products are released that promote the development of micropores and mesopores. The resulting hierarchical porosity, combined with the higher surface basicity (pH_PZC_ = 9.3), provides abundant active sites and favourable electrostatic conditions for the adsorption of positively charged metal ions. These effects are consistent with reports from the literature on KOH-activated biochar [39].

### 3.2. Nickel, Zinc, Copper and Benzotriazole Adsorption—Determination of Adsorbent Dose, Adsorption Time, and pH of the Solution

**Figure 5:**
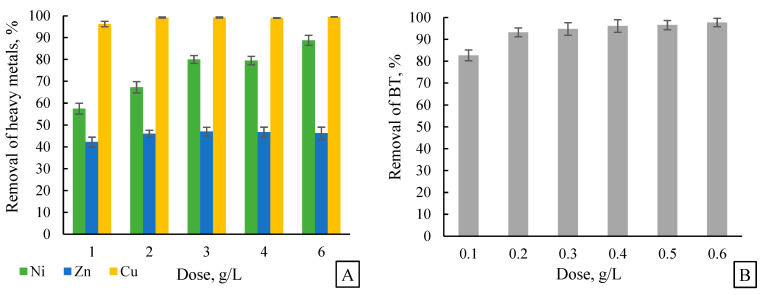

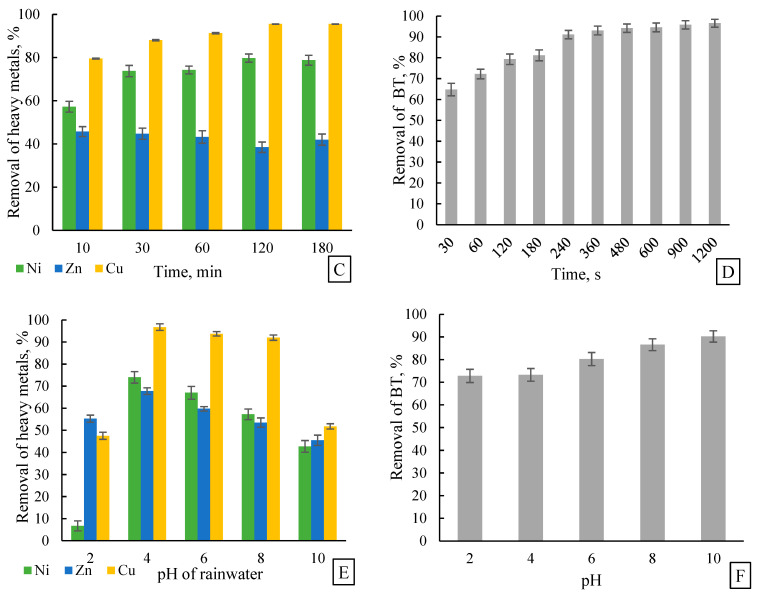
Effect of the adsorbent dose on the removal of zinc, copper, and nickel (**A**) and BT (**B**); Effect of adsorption time on the removal of metals (**C**) and BT (**D**); effect of the pH of the rainwater on the removal of metals (**E**) and BT (**F**). The initial metal concentration in raw water was 4 mg/L, and the initial BT concentration was 1 mg/L, mean ± SD (n = 3).

The aim of the study was to evaluate the effectiveness of the new biochar, obtained from the silkworm cocoon, in the process of adsorption of nickel (Ni), copper (Cu), and zinc (Zn) ions from rainwater. Based on LPNA analysis and SEM images, AC3 carbon was selected for further study. Analysis of the experimental results included the determination of optimal adsorption process parameters such as adsorbent dose, contact time, and water pH, which is crucial for the practical application of this material in water purification technology. In addition, data analysis allowed the determination of the adsorption isotherms and kinetics of the process, allowing for a deeper understanding of the mechanisms responsible for the removal of individual heavy metals. Figure 5 shows the relationship between biochar dose (A), adsorption time (B), and pH of the solution on the degree of removal of metals and benzotriazole from rainwater.

A higher dose of biochar increases the number of available adsorption sites, which generally leads to higher removal efficiency of heavy metals [40]. For copper and nickel, beyond a certain dose (4–6 g/L), further increases in efficiency are less pronounced, which may indicate that a substantial fraction of adsorption sites are occupied. However, it should be noted that this experiment, which varies the adsorbent dose at a fixed metal concentration, does not allow for a direct estimation of the maximum adsorption capacity of the biochar. Determining the true saturation point would require experiments with a fixed adsorbent dose and varying metal concentrations. For zinc, the effect of dose increase is less pronounced, possibly due to its lower affinity for active sites [41]. In practical water purification applications, the dose of adsorbent should be selected according to the type of dominant impurities. Adsorption occurs most rapidly in the initial phase (first 30 min) due to high site availability and slows after 60 to 120 min as the sites become increasingly occupied and equilibrium is approached.

Heavy metal adsorption is strongly dependent on the pH of the solution, which is due to the chemical properties of the metals and the surface of the adsorbent [42]. Due to the presence of KOH during activation, carbon can have a more alkaline surface area, which can mitigate the effect of competition under acidic conditions [43]. At low pH (e.g., 2 to 4), the concentration of H^+^ ions is very high, leading to competition with heavy metal ions for adsorption sites on the surface of biochar. The highest removal of Zn(II) and Ni(II) is observed in the pH range of 6 to 8, when zinc and nickel ions are converted to the hydroxyl form (e.g., Zn(OH)^+^, Ni(OH)^+^), which are more easily adsorbed on the surface of biochar [44]. This is also due to weakening of the competition of H^+^ ions and the possible precipitation of some metals in the form of hydroxides. Copper has a higher affinity for the surface of biochar, especially if the surface of the coal contains functional groups such as carboxyl or hydroxyl. At a high pH (above 8), the adsorption efficiency decreases, which may be due to excessive alkalinity, which affects the chemical stability of metals and their form in solution, since under more alkaline conditions they can precipitate as insoluble hydroxides, which reduces the adsorption efficiency.

As with heavy metals, the adsorption efficiency of benzotriazole in biochar depends on several factors, such as the adsorbent dose, contact time, and pH. Unlike metals, benzotriazole is an organic molecule and its interactions with biochar can occur through hydrophobic interactions, van der Waals forces, or electrostatic interactions [45]. To better understand the adsorption efficiency of benzotriazole under different conditions, it is useful to analyse the impact of these parameters on the degree of removal of benzothiazole. It has been observed that benzotriazole, as an organic compound, binds more easily to the micropores of biochar, making the process faster and require lower doses. An increase in dose from 0.01 g to 0.06 g results in an increase in removal from 82.71% to 97.76%. At higher doses, a saturation effect occurs, i.e., an excess of adsorbent does not significantly increase removal, because most of the benzotriazole has already been adsorbed. Regarding the kinetics of the process, the adsorption of metal ions is often slower because it requires the migration of hydrated cations to the active sites, which may involve both pore diffusion and ion exchange. This process can be hindered by the partial dehydration of the ions when entering narrow pores, resulting in the need for longer contact times to reach equilibrium. In contrast, benzotriazole is a neutral organic molecule under most environmental pH conditions and does not require dehydration to enter micropores, which can facilitate faster diffusion compared to hydrated metal ions of similar effective size. Similar observations were reported by Masson et al. [46], who found that the kinetics of adsorption tends to increase for adsorbates that can diffuse more readily diffuse into carbon micropores. In our study, benzotriazole adsorption was initially very rapid (30 s—64.76%, 120 s—79.29%), reaching removal of 94.55% after 600 s and only slightly increasing to 96.55% after 1200 s. The gradual decrease in the availability of active sites over time suggests that the process becomes limited by diffusion of intraparticles, as confirmed by the Weber–Morris model. Furthermore, changes in the pH of the solution indicated that at low pH the carbon surface becomes more positively charged, which may lead to the electrostatic repulsion of benzotriazole in its cationic form. At a higher pH, the carbon becomes more negatively charged, which favours the adsorption of benzotriazole, because at this pH, benzotriazole remains the basic form of neutral, co-creating its adsorption on the hydrophobic surface of the carbon.

### 3.3. Isotherm and Adsorption Kinetics

On the basis of the adsorbent dose studies, popular adsorption isotherm models such as the Langmuir model and Freundlich model were used to fit the experimental data. Table 3 shows the parameters estimated from the linear isothermal data plots.

**Figure 6:**
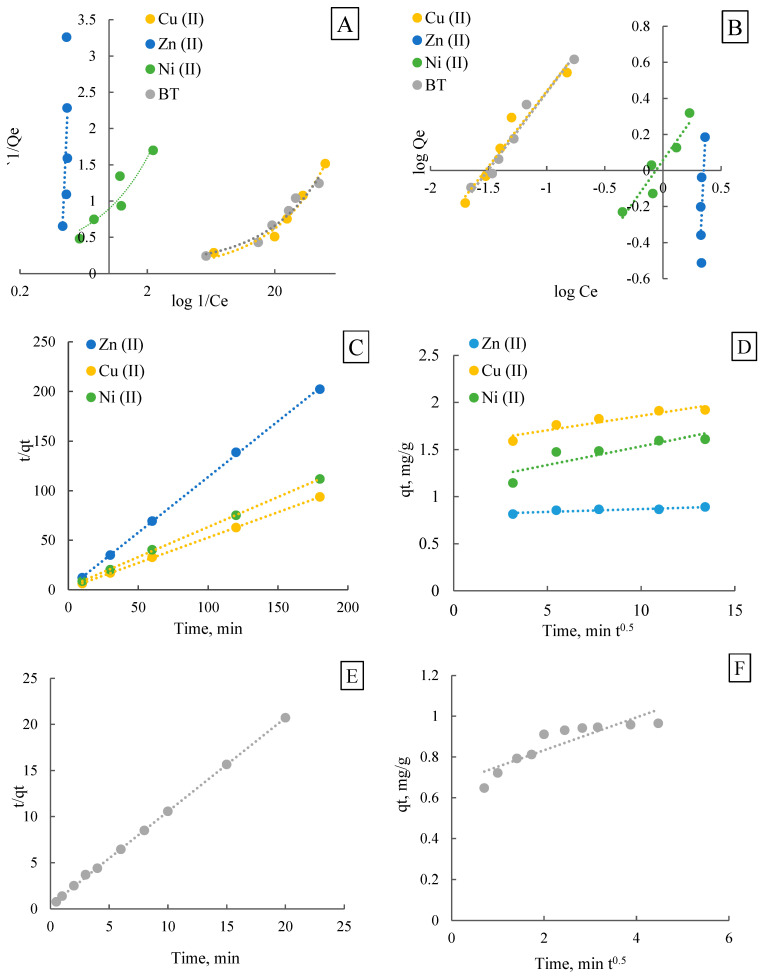
Adsorption isotherms and kinetic models: (**A**) Langmuir, (**B**) Freundlich, (**C**) pseudo-second order and (**D**) Weber–Morris models for Cu(II), Zn(II), and Ni(II), (**E**) pseudo-second order and (**F**) Weber–Morris models for benzotriazole.

On the basis of the calculations performed, it can be observed that different compounds show different fit to isotherm models, suggesting different adsorption mechanisms. In particular, the analysis of the Langmuir and Freundlich isotherms for heavy metals and benzotriazole allows for a better understanding of the interaction of these substances with the adsorbent (Figure 6A,B). For Cu(II), both Langmuir and Freundlich models exhibit high correlation coefficients (R^2^ = 0.98 and R^2^ = 0.95, respectively), indicating that the difference in fit is not sufficient to unequivocally determine surface homogeneity. This suggests that adsorption likely occurs on a heterogeneous surface with contributions from both monolayer and multilayer adsorption, which is typical of chemisorption, where copper ions interact with functional groups of the adsorbent (e.g., carbonyl, hydroxyl, carboxylic). For Zn(II), neither the Langmuir nor Freundlich model provides a satisfactory fit (R^2^ < 0.6), indicating a more complex or poorly defined adsorption behaviour [47]. This could be attributed to the low degree of adsorption, potential desorption, or competition with other ions. Weak interactions between Zn(II) and the surface could facilitate its return to the solution, which may also explain the anomalous values of Q_m_ and K_F_. For Ni(II), the adsorption process is slightly better described by the Freundlich model (R^2^ = 0.89) than by the Langmuir model (R^2^ = 0.87), indicating a more heterogeneous adsorption surface and the possibility of multilayer adsorption. This behaviour may be related to the smaller ionic radius of Ni(II), which could enable deeper penetration into micropores, in contrast to Cu(II), which tends to interact more strongly with surface functional groups. For benzotriazole, the results of the isothermal fit indicate the best fit to the Freundlich model, suggesting that the adsorption process takes place on a heterogeneous surface and may be multilayered. This means that benzotriazole does not bind specifically to specific active sites but instead decomposes on the surface of the adsorbent in a more dispersed manner [48]. This analysis indicates that adsorption behaviour is influenced not only by model parameters but also by chemical interactions between adsorbates and functional groups on the biochar surface. For metal ions, electrostatic attraction to negatively charged biochar surfaces (pH_PZC_ = 9.3) and complexation with surface functional groups such as the hydroxyl and carboxyl groups are likely responsible for the high removal efficiency. Benzotriazole adsorption is primarily driven by hydrophobic interactions, van der Waals forces, and potential π-π interactions with the carbon matrix. These interactions explain the differences in adsorption capacity and kinetics among the studied compounds. Figure 6C,D show pseudo-second-order kinetics and the Weber-Morris model for heavy metals, and Figure 6E,F shows benzotriazole.

The adsorption process of heavy metals and organic compounds on the surface of the adsorbent plays a key role in evaluating the purification efficiency of aqueous solutions. Adsorption kinetics provides important information on the mechanism of this phenomenon, the speed of the process and the factors influencing its course. The adsorption kinetics of Zn(II), Cu(II), and Ni(II) ions, as well as the benzotriazole compound, were analysed on the adsorbent. Based on the data obtained, parameters such as the adsorption rate constant and the C value of the Weber–Morris model were determined, allowing the evaluation of the dominant mass transport mechanisms (Table 4). The pseudo-first-order model cannot be applied correctly because the log(q_e_ − q_t_) values lead to an error (negative values inside the logarithm). This means that this model does not match the data [49].

Analysis of the adsorption kinetics of Cu(II), Zn(II), Ni(II) and benzotriazole was carried out on the basis of the pseudo-secondary model and the Weber-Morris model, which allow us to determine the mechanism of adsorption and the effect of diffusion on the process. The values of the parameters of the pseudo secondary kinetic model indicate that zinc 2.2076 g/(mg·min) has the highest adsorption rate K_2_, while benzotriazole 0.0217 g/(mg·min) has the lowest adsorption. However, the speed of adsorption alone is not the only factor determining the efficiency of the process. Knowing the value of the adsorption capacity at steady state Q_m_ allows us to assess how much adsorbate actually binds to the adsorbent surface. In this case, the highest Q_m_ was obtained for Cu(II) 1.906 mg/g, suggesting stronger copper-adsorbent interactions. This value is much higher than for Zn (II) 0.891 mg/g, although zinc shows a higher adsorption rate. This means that Zn (II) can form weaker bonds or adsorb mainly on the surface rather than penetrating the pores of the adsorbent. The nickel results show average values for both K_2_ = 0.4136 g/(mg·min) and Q_m_ = 1.539 mg/g, suggesting that nickel adsorption is neither the fastest nor the most efficient. The results indicated that the adsorption of metals and benzotriazole is differentiated, both in terms of the rate and mechanism of adsorption. To better understand the process, we conducted a further analysis using the Weber-Morris model to determine to what extent intraparticle diffusion contributes to the overall kinetics. The highest value of the internal diffusion constant K_wm_ was recorded for BT 0.08 mg/(g·min^0.5^), indicating a significant effect of diffusion on this process. The value of parameter C, which determines the effect of the boundary layer, was highest for Cu(II) 1.55 mg/g, and Ni(II) 1.14 mg/g, suggesting that adsorption is largely dependent on surface interactions in their case. Benzotriazole had the lowest value of C = 0.67 mg/g, indicating that its adsorption is more closely controlled by internal diffusion.

## 4. Comparison with Other Materials

In this chapter, the adsorption efficiency of silkworm cocoon biochar is compared with that of other materials used in the removal of contaminants, such as heavy metals and organic pollutants. Table 5 compares different adsorbents in terms of their adsorption efficiency, adsorption isotherm model, and specific surface area (SSA, m^2^/g). This analysis allows us to assess how silkworm cocoon biochar compares with other materials, considering both performance and physicochemical properties, which are crucial in the process of removing contaminants from rainwater.

The adsorption performance of silkworm cocoons, presented in Table 5, indicates that this material can achieve adsorption capacities of 3.5 mg/g for Cu, 2.1 mg/g for Ni, 1.53 mg/g for Zn, and 2.33 mg/g for benzotriazole. The material shows a clear selectivity toward Cu and organic pollutants. Importantly, its specific surface area (SSA) of 1614 m^2^/g is relatively high for a biochar and comparable to values typical of commercial activated carbons, helping to explain its adsorption behaviour. Silkworm cocoon waste is generated in regions with a developed silk industry and can serve as a sustainable raw material for biochar production. Although its availability may be locally limited, using this waste stream could provide both ecological and economic benefits.

## 5. Conclusions

In this study, the adsorption efficiency of biochar derived from silkworm cocoons for Cu(II), Ni(II), Zn(II), and benzotriazole in model rainwater was evaluated. The porous structure and adsorption efficiency were found to depend strongly on the pyrolysis temperature. Pyrolysis at 800 °C resulted in the formation of a highly developed microporous structure with a large surface area, whereas excessive heating at 1000 °C partially degraded the structure, reducing adsorption efficiency. Cu(II) adsorption was the most effective, reaching 99% even at low sorbent doses. For Ni(II), the adsorption efficiency was approximately 80% at a dose of 3 g/L, while Zn(II) adsorption was lower, approximately 47%. Isotherm and kinetic analyses revealed diverse adsorption mechanisms: Cu(II) and Ni(II) bound mainly through surface interactions, and benzotriazole adsorption was controlled by diffusion and multilayer processes, while Zn(II) showed the weakest affinity. The adsorption kinetics of all tested compounds followed the pseudo-second-order model. With a specific surface area comparable to that of commercial activated carbons, biochar from silkworm cocoons represents a promising use of silk industry residues for adsorbent production. The high removal efficiencies observed for Cu(II) (99%) and benzotriazole (93%) suggest that this material could potentially be applied in broader water treatment contexts, such as industrial wastewater or landfill leachates, although further studies are needed to confirm performance under real-world conditions.

The main advantages of the developed material include the utilisation of an abundant biological waste precursor, the high adsorption capacity for both metal ions and the organic contaminants, and structural properties comparable to those of commercial activated carbons. However, several practical limitations should be considered. The adsorption efficiency varied among target pollutants, with a lower performance for Zn(II), and the regeneration and reuse of the biochar have not yet been optimised. Moreover, although the precursor is an easily available waste, large-scale activation using KOH requires careful control of the process conditions and post-treatment to minimise chemical residues.

## Figures and Tables

**Table 1:** Physicochemical characteristics of the tested rainwater (mean value obtained from 5 rainwater samples taken at intervals of several days).

Parameter	Unit	Rainwater (Mean ± SD, n = 5)
pH	-	7.2 ± 0.20
Conductivity	µS/cm	364 ± 5.00
Colour	mgPt/L	37 ± 2.00
Hardness	mg/L	0.4 ± 0.05
TOC	mg/L	16.2 ± 0.80
COD	mg/L	108 ± 5.00
Zn	mg/L	1.74 ± 0.08
Cu	mg/L	0.03 ± 0.005
Ni	mg/L	0.25 ± 0.01
Pb	mg/L	0 ± 0.00

**Table 2:** Structural parameters of AC biochars (mean ± SD, n = 2).

Parameter	AC1	AC2	AC3	AC4
BET surface area, m^2^/g	1178 ± 10	856 ± 8	1614 ± 12	746 ± 7
Langmuir surface area, m^2^/g	1610 ± 14	1198 ± 10	2303 ± 18	1083 ± 12
Total DFT micropore volume, cm^3^/g	0.476 ± 0.008	0.35 ± 0.006	0.679 ± 0.01	0.315 ± 0.005
BJH cumulative mesopore volume, cm^3^/g	0.063 ± 0.002	0.09 ± 0.003	0.123 ± 0.004	0.105 ± 0.003

**Table 3:** Parameters of the Freundlich and Langmuir equations.

Parameter	Langmuir			Freundlich		
	Q_m_ (mg/g)	K_L_ (L/mg)	R^2^	K_F_ ((mg/g) L/mg)^n^	n	R^2^
**Cu (II)**	37.88	0.89	0.98	18.84	1.2	0.95
**Zn (II)**	0.06	0.42	0.35	1.59 × 10^−5^	0.07	0.58
**Ni (II)**	5.47	0.26	0.87	1.14	1.09	0.89
**BT**	9.01	3.99	0.95	19.40	1.17	0.96

**Table 4:** Parameters for the pseudo-second order kinetic and the Weber-Morris model for the adsorption of heavy metals and benzotriazole.

	Pseudo-Second-Order Equation Parameters			Weber-Morris		
Parameter	K_2_ (g/(mg·min))	Q_e_ (mg/g)	R^2^	K_wm_	C	R^2^
**Cu(II)**	1.04	1.91	0.99	0.03	1.55	0.88
**Zn(II)**	2.21	0.89	0.99	0.01	0.81	0.82
**Ni(II)**	0.41	1.54	0.99	0.04	1.14	0.76
**BT**	0.02	0.98	0.99	0.08	0.67	0.78

**Table 5:** Comparison of the adsorption efficiency of different materials (including the silkworm cocoon) in the removal of contaminants.

Adsorbent	Pollution	Adsorption Efficiency/ Adsorption Capacity	Model of Isotherm	SSA (m^2^/g)	Ref.
Biochar from silkworm cocoons	Benzotriazole Ni, Cu, Zn.	99% Cu 3.5 mg/g	Langmuir/Freundlich (Cu)	1614	Own research
		80% Ni 2.1 mg/g			
		47% Zn 1.53 mg/g	Freundlich (Ni and BT)		
		93% BT 2.33 mg/g			
Biochar of Scots Pine Biochar of Silver Birch	Cd,	Pb 1.29–3.77 µg/g	Freundlich	10	[50]
	Pb,	2.37–4.49 µg/g			
	Cu,	Cu 128.7 µg/g		7	
	Zn	Zn 107.0 µg/g			
Granular activated carbon (WG-12)	Cd, Cu, Zn Acenaftin Fenantgard	Acenaftyna 0.31–2.63 mg/g	Langmiur	1010	[51]
		Fenantgard 0.4–7.36 mg/g			
TiO 2	Pb, methylene blue	High adsorption capacity	Langmiur	417	[52]
UIO-66 (MOF)	Pb	381.195 mg/g	Langmuir	n.d.	[53]
UIO-66 (MOF)	methylene blue	91 mg/g	Langmiur	657, 906	[54]
Coconut shell activated carbon	Fe	9.67 mg/g	Freundlich	n.d.	[55]
	Pb	10.04 mg/g			
Natural zeolite	Cd	25.9	Langmuir/Freundlich	n.d.	[56]
	Cu	14.3 mg/g			
Biochar, RBC: rice husk, WBC: wood chips, MBC: mix	Cd	Cd > 9.15 mg/g;	n.d.	n.d	[57]
	Pb	Pb > 9.98 mg/g;			
	Zn	Zn > 6.58 mg/g;			
Graphene	Tetracyclin (TC), Cd, As	252 mg/g TC,	Freundlich	148	[58]
		234 mg/g Cd			
		14 mg/g As			
Carbon nanotubes	Cu, Co, Cd, Zn, Mn, Pb	Cu 3.49 mg/g	Freundlich	40–600	[59]
		Co 2.60 mg/g			
		Pb 2.96 mg/g			
Clay-cellulose biocomposite (CCB)	Pb	389.78 mg/g	Langmuir	n.d.	[60]
	Cd	115.96 mg/g			

## Data Availability

The original contributions presented in this study are included in the article. Further inquiries can be directed to the corresponding author.
